# In Silico Identification and In Vitro Evaluation of Natural Inhibitors of *Leishmania major* Pteridine Reductase I

**DOI:** 10.3390/molecules22122166

**Published:** 2017-12-06

**Authors:** Fabian C. Herrmann, Nirina Sivakumar, Joachim Jose, Maria P. Costi, Cecilia Pozzi, Thomas J. Schmidt

**Affiliations:** 1 Institute of Pharmaceutical Biology and Phytochemistry (IPBP), University of Muenster, PharmaCampus, Corrensstrasse 48, D-48149 Muenster, Germany; f_herr01@uni-muenster.de (F.C.H.); nirinasiva@gmail.com (N.S.); 2 Institute of Pharmaceutical and Medicinal Chemistry, University of Muenster, PharmaCampus, Correnstrasse 48, D-48149 Muenster, Germany; joachim.jose@uni-muenster.de; 3 Department of Life Sciences, University of Modena and Reggio Emilia, Via G. Campi 103, 41125 Modena, Italy; mariapaola.costi@unimore.it; 4 Department of Biotechnology, Chemistry and Pharmacy, University of Siena, Via A. Moro 2, 53100 Siena, Italy; pozzi4@unisi.it

**Keywords:** *Leishmania major*, cutaneous Leishmaniasis, pteridine reductase I inhibitor, natural product, in silico screening

## Abstract

In a continuation of our computational efforts to find new natural inhibitors of a variety of target enzymes from parasites causing neglected tropical diseases (NTDs), we now report on 15 natural products (NPs) that we have identified as inhibitors of *Leishmania major* pteridine reductase I (*Lm*PTR1) through a combination of in silico and in vitro investigations. Pteridine reductase (PTR1) is an enzyme of the trypanosomatid parasites’ peculiar folate metabolism, and has previously been validated as a drug target. Initially, pharmacophore queries were created based on four 3D structures of *Lm*PTR1 using co-crystallized known inhibitors as templates. Each of the pharmacophore queries was used to virtually screen a database of 1100 commercially available natural products. The resulting hits were submitted to molecular docking analyses in the substrate binding site of the respective protein structures used for the pharmacophore design. This approach led to the in silico identification of a total of 18 NPs with predicted binding affinity to *Lm*PTR1. These compounds were subsequently tested in vitro for inhibitory activity towards recombinant *Lm*PTR1 in a spectrophotometric inhibition assay. Fifteen out of the 18 tested compounds (hit rate = 83%) showed significant inhibitory activity against *Lm*PTR1 when tested at a concentration of 50 µM. The IC_50_ values were determined for the six NPs that inhibited the target enzyme by more than 50% at 50 µM, with sophoraflavanone G being the most active compound tested (IC_50_ = 19.2 µM). The NPs identified and evaluated in the present study may represent promising lead structures for the further rational drug design of more potent inhibitors against *Lm*PTR1.

## 1. Introduction

In continuation of our combined in silico/in vitro studies to find natural products inhibiting vital target enzymes of protozoan parasites [1,2], we have now focused on *Leishmania major* pteridine reductase I (*Lm*PTR1), which has previously been suggested as a drug target [3]. As the etiological agents of several neglected tropical diseases (NTDs), parasites of the group Trypanosomatidae, including such of the genus *Leishmania*, are responsible for a significant disease burden, especially for rural populations in tropical regions living under poor socioeconomic conditions. Leishmaniasis is caused by various species of *Leishmania*, which lead to different clinical forms of the disease. It threatens the health of nearly one billion people worldwide [4]; an annual number of about 700,000 to one million cases are reported according to the World Health Organization [5]. As such, CL represents a global health problem of high relevance [5]. *Leishmania major* (*Lm*) is one of the species causing cutaneous Leishmaniasis (CL). CL is characterized by an often subacute progression, and while responsible for a relatively low rate of fatality, this most common form of *Leishmania* infection leads to severe and disfiguring skin lesions [6]. 

*Lm*, as all *Leishmania* species, is an intracellular parasite; as a result, the research for vaccines has not been successful, and chemotherapeutics are the only option for fighting the disease [7]. Problematically, all of the available compounds for the treatment of Leishmaniasis, and CL in particular, suffer from severe drawbacks such as high toxicity, lack of efficacy, or the need for hospitalization [5]. Additionally, their mechanisms of action are mostly unknown, and shortcomings concerning their administration, such as intravenous and long-lasting therapy regimes, additionally hamper therapy in rural and underdeveloped populations. These circumstances are severely limiting the current therapy of CL, underlining the urgent need for innovative chemotherapeutic options to sufficiently treat CL.

Due to fundamental phylogenetic differences between mammals and members of the Trypanosomatidae group, several metabolic pathways and their corresponding enzymes have been identified as potential targets for antileishmanial therapies in the past decades [8]. In particular, the peculiar folate metabolism of the *Leishmania* species has increasingly attracted interest as a promising starting point for innovative therapies [9,10]. Although inhibitors of the dihydrofolate reductase (DHFR, catalyzing the hydration of folic acid to di- and tetrahydro folic acid) are successfully used in therapy, e.g., malaria [11], *Leishmania* species show resistance against common antifolates such as methotrexate (MTX). Pteridine reductase I (PTR1), an oxidoreductase unique to kinetoplastids, is considered responsible for this DHFR resistance because it allows the parasites to produce reduced folates in an alternative pathway, thus compensating for the inhibition of DHFR. Under physiological conditions, PTR1 contributes about 10% to the production of the needed folate equivalents [12]. In the course of reduced DHFR activity, a PTR1 upregulation can be observed in members of the genus *Leishmania*, rendering these parasites resistant to this therapeutic strategy [12]. Targeting PTR1, especially in combination with DHFR inhibition, could therefore lead to effective and innovative antileishmanial chemotherapies. At present, however, no inhibitors of leishmanial PTR1 are available as therapeutic options.

In the present study, we report on the identification of several natural products (NPs) as inhibitors of *Leishmania major* pteridine reductase I (*Lm*PTR1) by in silico screening (pharmacophore-based virtual screening and docking simulations) followed by experimental evaluation of the resulting hits in a spectrophotometric inhibition assay with recombinant *Lm*PTR1.

## 2. Results

### 2.1. In Silico Identification of Natural Products as Potential LmPTR1 Inhibitors

A database of commercially available natural products supplied by PhytoLab GmbH & Co. KG (Vestenbergsgreuth, Germany; http://phyproof.phytolab.de/), which consisted of 1100 entries, was used as compound collection for the in silico prediction of possible inhibitors against *Lm*PTR1 by means of pharmacophore-based virtual screening followed by docking simulations. All of the computations were performed with the software package MOE [13]. Initially, compounds not likely to have drug-like properties were eliminated from the database employing Lipinski’s rule of five [14], resulting in a final database size of 737 NPs. 3D models of the remaining structures were generated and subjected to geometry optimization by means of a conformational search. A maximum of the 10 energetically most favorable conformers of each compound were stored for subsequent virtual screening.

**Figure 1:**
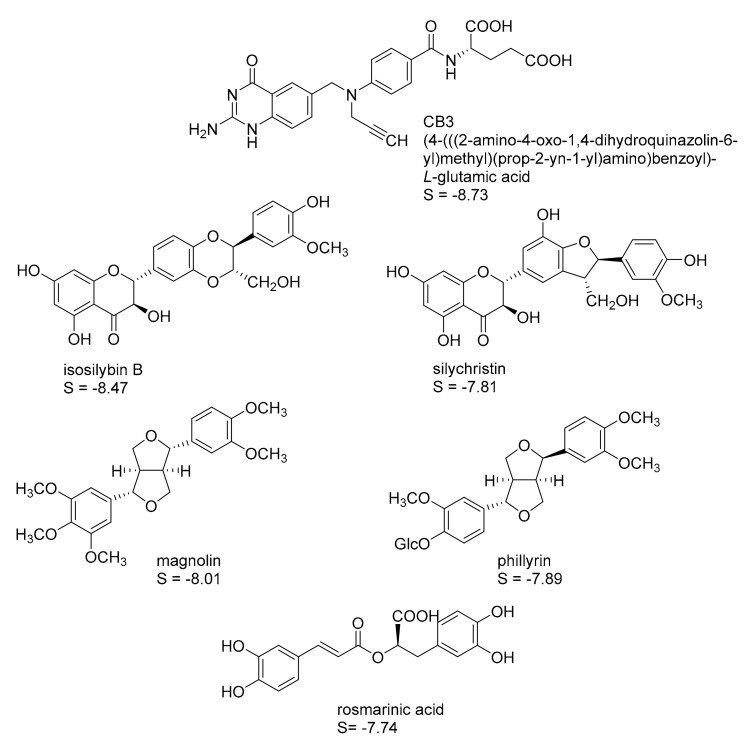
Best five hits from the virtual screening based on the CB3 pharmacophore with the *Leishmania major* pteridine reductase I (*Lm*PTR1) Protein Data Bank (PDB) identification (ID) “2BFA”; S-scores of the induced-fit docking simulations are given in kcal/mol. A “self-docking” computation of the co-crystallized inhibitor CB3 was performed accordingly.

**Figure 2:**
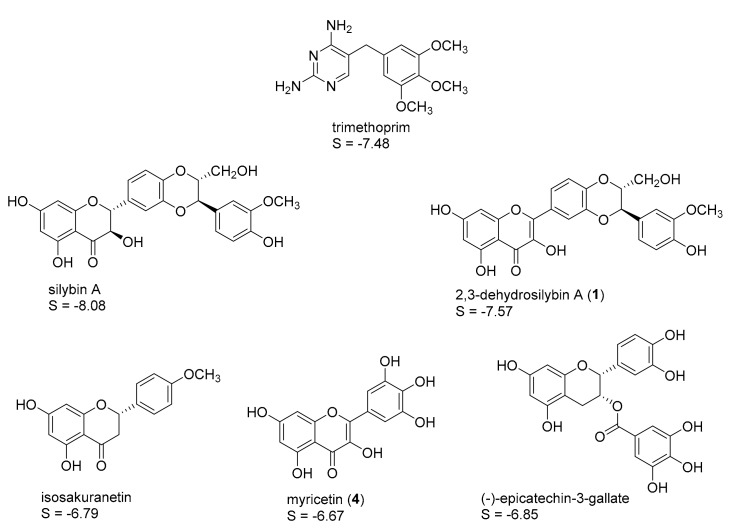
Best five hits from the virtual screening based on the trimethoprim pharmacophore (*Lm*PTR1 PDB-ID “2BFM”); S-scores of the induced-fit docking simulations are given in kcal/mol. A “self-docking” computation of the co-crystallized inhibitor trimethoprim was performed accordingly.

**Figure 3:**
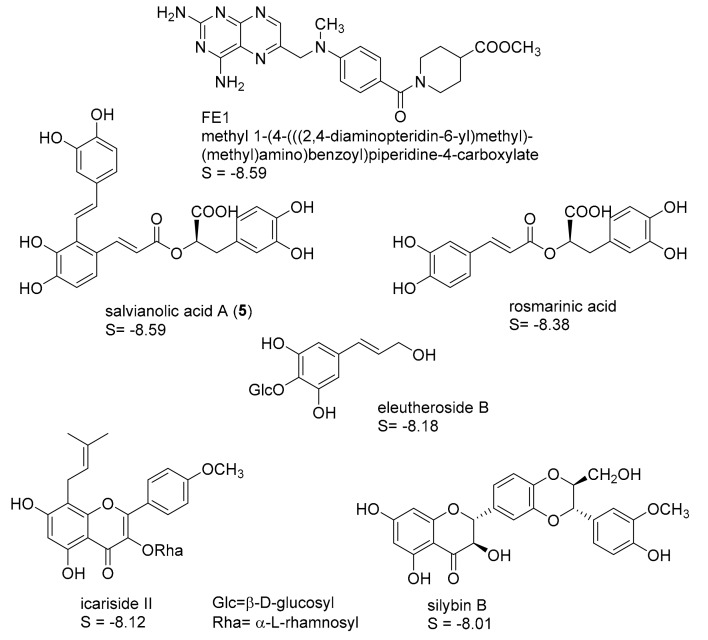
Best five hits from the virtual screening based on the FE1 pharmacophore (*Lm*PTR1 PDB-ID “2QHX”); S-scores of the induced-fit docking simulations are given in kcal/mol. A “self-docking” computation of the co-crystallized inhibitor FE1 was performed accordingly.

**Figure 4:**
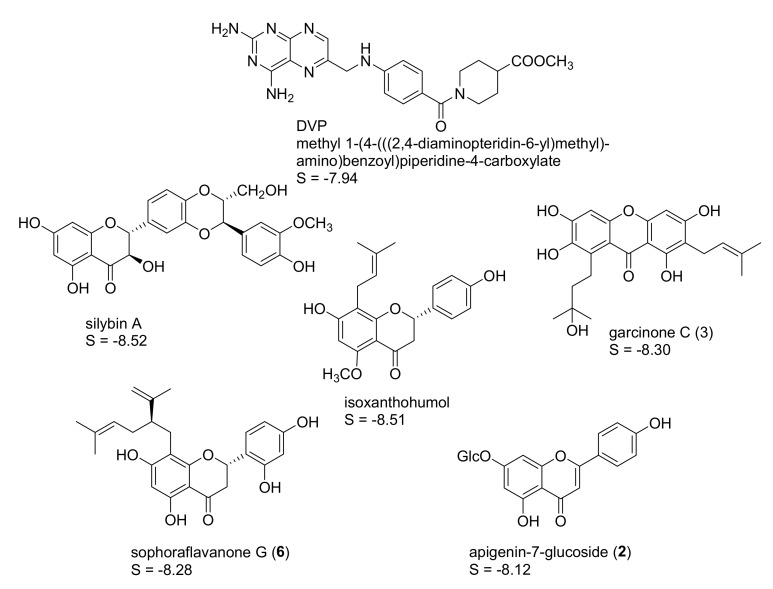
Best five hits from the virtual screening based on the DVP pharmacophore (*Lm*PTR1 PDB-ID “3H4V”); S-scores of the induced-fit docking simulations are given in kcal/mol. A “self-docking” computation of the co-crystallized inhibitor DVP was performed accordingly.

Four protein structures of pteridine reductase 1 from *Leishmania major*, each co-crystallized with a molecule of the oxidized co-substrate nicotinamide adenine dinucleotide (NADP^+^) and an inhibitor molecule, were retrieved from the Protein Data Bank (PDB) [15] (PDB-IDs are reported in the experimental section) and subjected to structural correction and energy minimization. The co-crystallized inhibitors (see Figure 1, Figure 2, Figure 3 and Figure 4) of the four protein models elucidated by X-ray crystallography subsequently served as templates for the creation of four independent pharmacophore queries (see Supplementary Materials Figures S1–S4; for a detailed description of the pharmacophore generation, see Section 3.4). Each of the four pharmacophores was then used as a virtual screening filter for the NPs database yielding hit collections, which were subsequently docked into the corresponding inhibitor binding site of the protein structure from which the respective pharmacophore query had been obtained, employing a fast rigid docking algorithm. The resulting docking poses were ranked by their docking scores (S-scores, in kcal/mol; more negative scores indicating higher binding affinity). The 10 compounds with the most negative S-scores were selected in each case for a more refined docking simulation (i.e., induced fit docking algorithm, allowing flexibility for the docked ligand as well as the binding site). Five compounds with the most negative S-scores in the induced fit docking with each protein structure were considered promising candidates for in vitro evaluation. Figure 1, Figure 2, Figure 3 and Figure 4 show these best hits obtained for each of the four pharmacophores/protein structures, as well as the respective co-crystallized inhibitors used to construct the pharmacophore queries. The calculated S-scores (given in kcal/mol) are also reported in Figure 1, Figure 2, Figure 3 and Figure 4.

It is interesting to note that despite the considerable structural diversity of the investigated NPs (including many non-aromatic compounds such as terpenoids of various sizes), all of the selected hits are aromatic compounds. Most of them are flavonoids, but two lignans, some caffeic acid esters, one xanthone, and one monomeric phenylpropanoid are also included. Two compounds (silybin A and rosmarinic acid) were among the best five hits in two of the four cases, so that in total, 18 NPs were obtained from the manufacturer for experimental evaluation.

### 2.2. In Vitro Evaluation of the Identified NPs against LmPTR1

In order to assess the inhibitory activity of the compounds identified in silico, spectrophotometric inhibition assays were performed on recombinant *Lm*PTR1, measuring the decreasing concentration of NADPH at 340 nm as a linear kinetic parameter for *Lm*PTR1 activity at saturating concentrations of NADPH (co-substrate) and folic acid (substrate). First, each in silico hit was added to a concentration of 50 µM, and the resulting linear plots of absorbance vs. time were compared with those recorded in the absence of an inhibitor. Of the 18 compounds predicted in silico as potential inhibitors, 15 indeed showed significant inhibitory activity at 50 µM (hit rate = 83%). The results of the in vitro assays are shown in Table 1. Six compounds, namely 2,3-dehydrosilybin A (**1**; a flavonolignan from *Silybum marianum*, Asteraceae [16]), apigenin-7-glucoside (**2**; a glycosylated flavone occurring e.g., in *Matricaria recutita*, Asteraceae [17]), garcinone C (**3**; a xanthone derivative naturally found in *Garcinia mangostana*, Clusiaceae [18]), myricetin (**4**; a flavonol occurring widespread in plants, e.g., in *Vitis vinifera*, Vitaceae [19]), salvianolic acid A (**5**; a caffeic acid derivative from *Salvia* species, Lamiaceae [20]) and sophoraflavanone G (**6**; a flavanone isolated e.g., from *Sophora flavescens*, Fabaceae [21]) inhibited the target enzyme by more than 50% at 50 µM. For these most active inhibitors, concentration-effect curves were determined, from which IC_50_ values could be determined in five cases. For 2,3-dehydrosilybin A (**1**), complete inhibition could not be achieved. The maximum of inhibition achieved with this compound was 64% at a concentration of 50 µM. This turned out to be due to the limited solubility of **1** under the assay conditions, so that in this case, an EC_50_ value was determined. Plots of the inhibitory activity as a function of concentration for compounds **1** to **6** are shown in the Supplementary Materials Figures S10–S15, and the resulting IC_50_ and EC_50_ values are reported in Table 1.

Five of the most active NPs (**1**–**4** and **6**) identified are structurally related to some extent, sharing a chromane (**3** and **6**) or chromene (**1**, **2** and **4**) system as a common skeletal element. These moieties are partially comparable to the pteridine system of the natural substrate folic acid, rendering these compounds promising initial hits for further inhibitor development and optimization. These findings are in agreement with recently published data from Borsari et al. [22], who also identified flavonoid derivatives as inhibitors of *Lm* as well as *Tb*PTR1 by a combined target-based phenotypic screening based on a library of natural products.

### 2.3. Mechanistic Considerations

**Figure 5:**
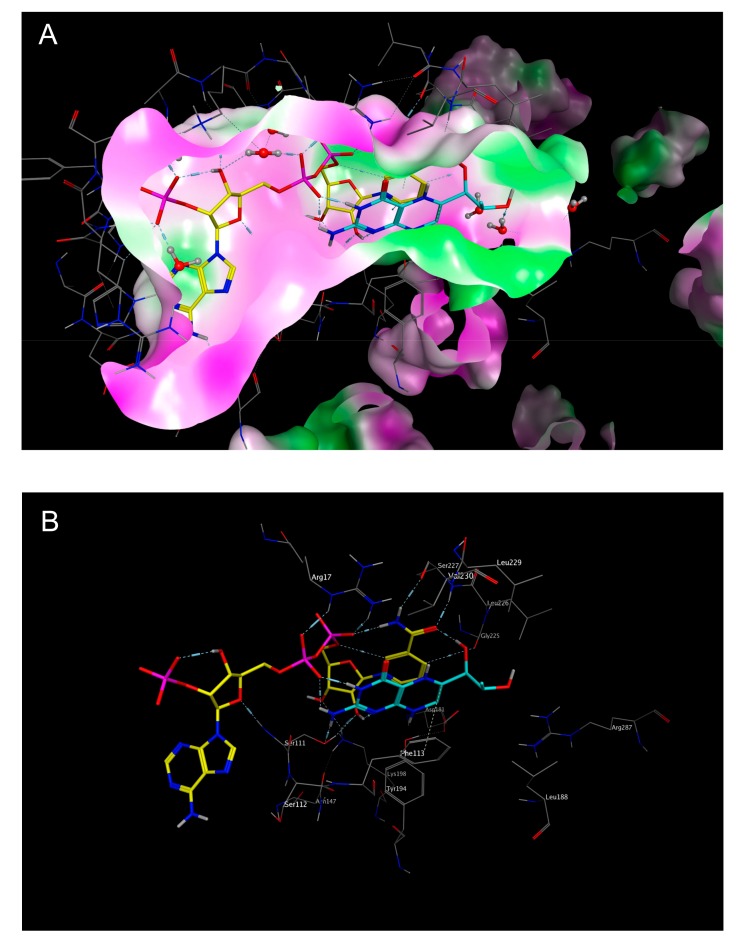
Representation of the catalytic site of *Lm*PTR1 (PDB-ID “2BF7”), with the co-crystallized nicotinamide adenine dinucleotide (NADP^+^) shown in yellow, and the co-crystallized substrate dihydrobiopterin (DHB) (7,8-dihydrobiopterin) shown in cyan. (**A**) The molecular surface of the binding site was colored according to lipophilicity, with green indicating high lipophilicity, and purple indicating low lipophilicity. Note the π-stacking interaction of the pteridine system with the nicotinamide moiety of NADP^+^, as well as the formation of H-bonds with one phosphate group of the co-crystallized co-substrate; (**B**) Surface not shown, but amino acid residues labeled.

The substrate-binding domain of *Lm*PTR1 is characterized by a considerable degree of lipophilicity, especially in a part mainly made up by hydrophobic amino acids like Tyr, Phe, Leu, or Val. The NADPH/NADP^+^-binding part of the catalytic site, on the other hand, is characterized by more hydrophilic amino acids and more polar properties overall. Due to the close vicinity of the co-substrate and substrate binding sites, the co-substrate NADPH/NADP^+^ may also contribute to the properties of the substrate cavity, introducing the possibility of polar interactions with a ligand bound in the folic acid binding site. Up to the end of this study, there were no crystal structures of *Lm*PTR1 available in complex with the substrate folic acid. In order to allow comparative analyses between the calculated docking poses of the NP inhibitors identified in this study, a protein structure model with co-crystallized dihydrobiopterin (DHB; PDB-ID “2BF7” [23]) was also investigated. DHB is substrate of PTR1 [24], and a structural analog of folic acid, with which it shares the pteridine system as the most important structural feature, and may hence allow at least a partial comparison to the expected binding mode of the natural ligand. Figure 5 shows the folic acid binding site of PDB-ID “2BF7” in complex with DHB and NADP^+^.

**Figure 6:**
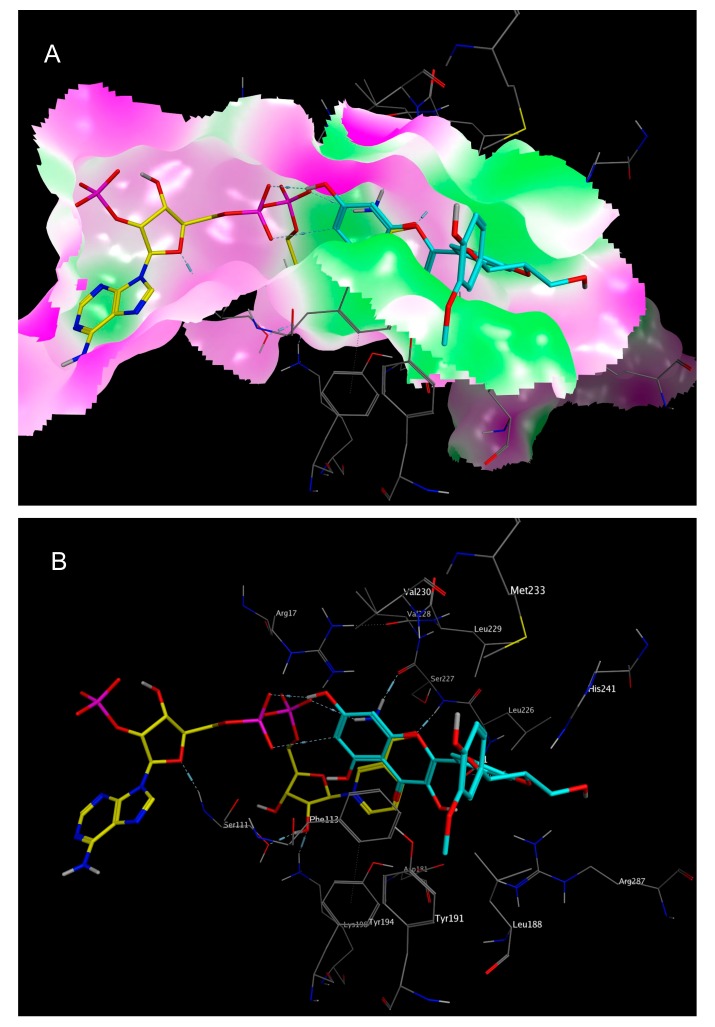
Best calculated docking pose of 2,3-dehydrosilybin A (**1**) in the folic acid binding site of *Lm*PTR1 (PDB-ID “2BFM”), with the co-crystallized NADP^+^ shown in yellow, and the best docking pose of **1** shown in cyan. (**A**) The molecular surface of the binding site was colored according to lipophilicity, with green indicating high lipophilicity, and purple indicating low lipophilicity; (**B**) Surface not shown, but amino acid residues labeled.

**Figure 7:**
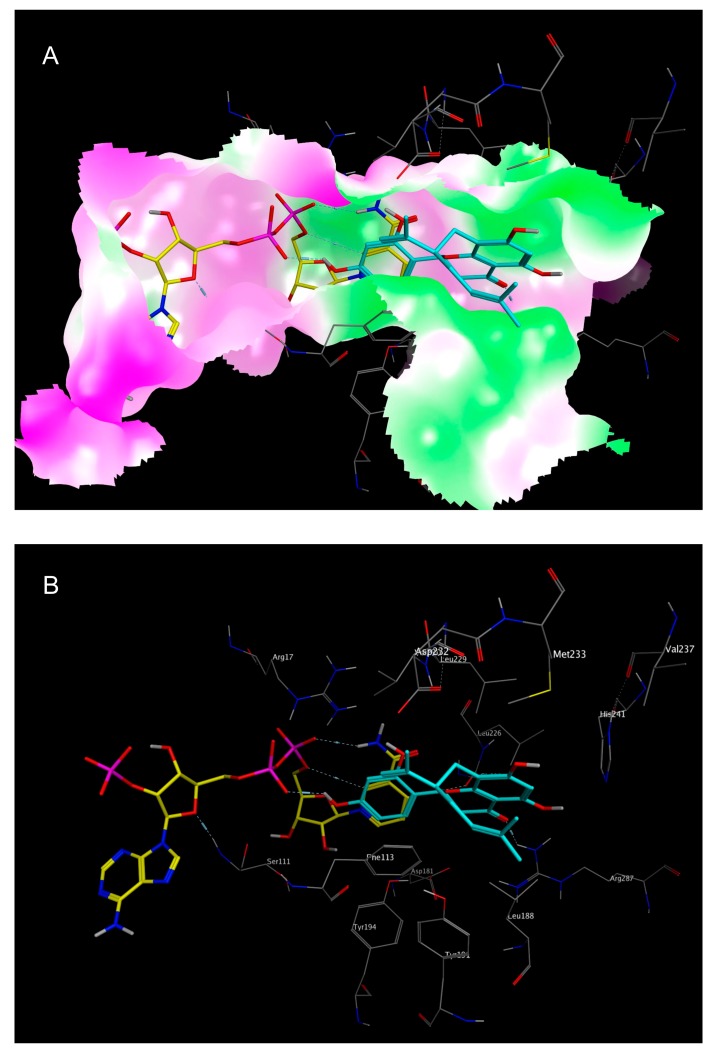
Best calculated docking pose of sophoraflavanone G (**6**) in the folic acid binding site of *Lm*PTR1 (PDB-ID “3H4V”), with the co-crystallized NADP^+^ shown in yellow, and the best docking pose of **6** shown in cyan. (**A**) Rendered surfaces are colored according to lipophilicity, with green indicating high lipophilicity, and purple indicating low lipophilicity; (**B**) Surface not shown, but amino acid residues labeled.

The docking simulations of all six NPs identified as most active (**1**–**6**, see Figure 6 and Figure 7 as well as Supplementary Materials Figures S5–S8) revealed favorable binding modes in the folic acid cavity of *Lm*PTR1, fitting the available space very well in each case. It is interesting to note that the best docking poses of all six NPs show some similarity with the experimentally determined binding mode of DHB (PDB-ID “2BF7”, Figure 5). In the case of DHB, the pteridine moiety is bound near the nicotinamide partial structure of NADP^+^, and presents a π–π interaction with the pyrimidine heterocycle of the co-substrate. Additionally, two hydrogen bonds are formed between both, the amino group at C-3 and the NH in position 2 of the pteridine heterocycle with a phosphate group of NADP^+^. This interaction profile including both the π–π interaction with the nicotinamide moiety and the H-bond formation with one phosphate group of NAPD^+^ can also be observed in each of the best docking poses of inhibitors **1**–**6** (Figure 6 and Figure 7 show the best docking poses for the two most active inhibitors, **1** and **6**, respectively. Analogous representations of compounds **2**–**5** are presented in the Supplementary Materials information, Figures S5–S8), indicating that these interactions are of considerable importance for the rational design of compounds inhibiting *Lm*PTR1.

It is interesting to note that apart from salvianolic acid A (**5**), the most active inhibitors share structural features with certain similarities, such as engaging in π–π interactions with the nicotinamide moiety, namely, a flavone (**2**), a flavonol (**1**, **4**), a flavanone (**6**) or a four-chromanone system (**3**). These partially aromatic ring systems are to some extent comparable to the pteridine structure of the natural substrate folic acid and were predicted in the docking simulations to display a very similar binding mode as the dihydropteridine part of DHB (e.g., in *Lm*PTR1, PDB-ID “2BF7”) by means of the formation of a π–π stacking interaction with the nicotinamide moiety of the co-substrate. The mentioned interactions were also predicted in silico for **5**, but are in this case mediated by one of the dihydroxybenzene moieties.

It is also worth mentioning that due to the lipophilic character of the substrate binding site of *Lm*PTR1, hydrophobic substituents could improve the inhibitory activity of NPs against *Lm*PTR1. In the cases of sophoraflavanone G (**6**; see Figure 7) and garcinone C (**3**; see Figure S6), lipophilic prenyl side chains were present, which were not surprisingly placed by the docking algorithm in the most lipophilic part of the folic acid binding site. For **3** and **6,** the in silico postulated position of their lipophilic side chains is very similar, with both of them lying in the vicinity of Leu 188 and Leu 226. Thus, the hydrophobic interactions of these structural elements may contribute to the *Lm*PTR1 inhibitory activity of **3** and **6**.

## 3. Materials and Methods

### 3.1. In Silico Modelling

All in silico operations, i.e., database and structure optimization, protein preparation, pharmacophore generation, and virtual screening, as well as all docking simulations, were performed with the Molecular Operating Environment (MOE), software version 2011.10 [13].

### 3.2. Database Design

The NP database used for the present study consisted of 1100 natural compounds of various origins, as available from PhytoLab GmbH & Co. KG (Vestenbergsgreuth, Germany; http://phyproof.phytolab.de/). The database entries were filtered according to Lipinski’s rule of five [14], and non-drug-like compounds (i.e., structures violating more than one of Lipinski’s rules) were discarded. 3D models of the remaining 737 compounds were generated and subjected to geometry optimization using the force field MMFF94x and low mode molecular dynamics conformational search (LowModeMD), in order to determine the most favorable conformations. A maximum of 10 low energy conformations of each NP within an energy window of 3 kcal/mol above the lowest energy conformer were collected in a new database, which served as compound collection for the subsequent virtual screening approach.

### 3.3. Acquisition and Preparation of the Protein’s 3D-Structure

Five 3D structures of pteridine reductase 1 from *Leishmania major* were retrieved from the Protein Data Bank (PDB-IDs “2BF7”, “2BFA”, “2BFM”, “2QHX”, and “3H4V”). The structures were subsequently corrected (with the structure preparation in MOE correcting terminal amino acids and protonation states, as well as faulty or misassigned amino acids) and energy was minimized using the MMFF94x force field [25] (an iterative minimization was employed, i.e., a series of minimizations were performed tethering heavy atoms with force constants ranging from 100 to 0 (100, 10, 1, 0.1, and 0)). All further steps were carried out with the fully relaxed protein structures containing, in each case, the co-crystallized co-substrate NADP^+^ and an inhibitor molecule, as well as a variable number of water molecules.

### 3.4. Pharmacophore Design and Virtual Screening

Based on the co-crystallized inhibitors of the four protein models “2BFA”, “2BFM”, “2QHX”, and “3H4V”, pharmacophore queries were created in order to perform virtual screenings on the natural product database. Initially, the interactions between the enzyme and the co-crystallized inhibitors in the active site were analyzed by creating an interaction table based on the “ligand interactions” feature implemented in MOE. Every interaction yielding a calculated S-score of less than or equal to −1 kcal/mol was considered to be of relevance for the inhibitors’ binding, and was therefore included into the pharmacophore query as a feature sphere. The radii of the feature spheres ranged from 1 to 2 Å, depending on the represented moiety (e.g., aromatic rings around 2 Å, and H-bond donors and acceptors around 1 Å, as suggested by MOE). Additionally, the surface of the binding site was also analyzed in order to detect potential further interaction sites not already addressed by the co-crystallized inhibitor. To achieve this, surface representations of the active site were calculated (e.g., through the electrostatic maps feature implemented in MOE), and potential further interactions of interest were included as additional feature spheres. The queries thus generated comprised five to seven features. Additionally, so-called “exclusion spheres” were added as features for every atom of the protein (radius of 1.42 Å, solvent molecules excluded) to rule out compounds that might be in agreement with the pharmacophore features, but would collide with the protein’s amino acids. The pharmacophore queries thus obtained are depicted in Supplementary Materials Figures S1–S4 (exclusion spheres not shown). Each of the queries was then used to virtually screen the NP database. In order to achieve a hit rate suitable for further in silico and in vitro analyses, the mentioned queries were only partially applied to a predefined extent (“partial match” feature in MOE), generating hit rates between 10 and 50 compounds for each pharmacophore, which were then collected into new databases and subsequently submitted to docking simulations.

### 3.5. Docking Simulations

The hits of each pharmacophore screening were submitted to molecular docking simulations. In order to ensure a valid docking protocol for each protein structure, the respective co-crystallized inhibitors were subjected to a self-docking simulation in the induced fit mode (i.e., both the ligand and the amino acid side chains in the docking site were allowed to change their geometry in order to achieve an optimal fit). In all of the cases, good reproducibility of the co-crystallized inhibitor conformation (Root mean square (RMS) deviation after superposition of the best calculated docking pose and the experimental co-crystallized conformation <1 Å) was achieved. These self-docking experiments also yielded docking scores (S-scores in kcal/mol) for the known inhibitors, which could then be compared with those of the natural product hits (see below). The hits from the four databases that resulted from the virtual screenings described above were all docked into the respective inhibitor binding sites of the four protein structures by means of a rigid docking algorithm (i.e., allowing flexibility only of the investigated compounds, and not of the binding pocket). The 10 docking poses of each molecule from each series were collected into new databases and ranked by their S-scores. The 10 compounds yielding the most negative S-scores were then submitted to an induced-fit docking simulation, conceding flexibility to the investigated ligands as well as the corresponding binding sites. The resulting most favorable docking poses and their calculated S-scores were again collected into new databases and ranked by their S-scores. The five NPs with the most favorable docking scores in each of the four series were selected as candidates for experimental evaluation by an in vitro enzyme inhibition assay.

### 3.6. Tested Compounds

All of the tested natural products were generously donated by PhytoLab GmbH & Co. KG (Vestenbergsgreuth, Germany).

### 3.7. Transformation of E. coli Cells (BL21 (DE3)) with Vector System LmPTR1-pET-15b

The pET-15b vector system containing the poly-His-*Lm*PTR1 gene together with a carbenicillin resistance selection marker was previously described [22]. Initially, the supplied vector was heat shock-transformed into competent *E. coli* cells (BL21 (DE3)). To do so, an aliquot of BL21 (DE3) cells was incubated together with 1–2 ng of plasmide at 42 °C for 90 s, followed by cooling on ice for 1 min and the subsequent addition of 1 mL of SOC medium (Super Optimal Catabolite medium, containing 20 g/L tryptone/peptone, 5 g/L yeast extract, 0.5 g/L NaCl, 2.5 g/L KCl, 20 mM d-glucose, and 10 mM MgCl_2_). After 1.5 h of incubation (37 °C, 200 rpm), 200 µL of the culture were transferred to an agar plate containing carbenicillin (50 µg/mL) and incubated overnight at 37 °C. A single culture was afterwards randomly selected and plated on another carbenicillin agar plate. After incubation overnight, the complete bacterial growth was collected using a sterile cotton swab and PP medium (proteose peptone medium, containing 15 g/L peptone, 5 g/L NaCl, 1 g/L Zulkowsky starch, 1 g/L KH_2_PO_4_, 0.8 g/L K_2_HPO_4_, and 20% (*v*/*v*) glycerol) was added. The yielded suspension was afterwards transferred to a sterile vessel filled with glas cryoperls, rapidly frozen with an ethanol/dry ice mixture, and stored at −80 °C.

### 3.8. Recombinant Expression and Purification of LmPTR1

For the heterologous expression of *Lm*PTR1, an overnight culture of the transformed *E. coli* BL21 (DE3) cells mentioned above was used to inoculate 2 L Erlenmeyer flasks filled with 400 mL of an LB medium containing carbenicillin (50 µg/mL carbenicillin), which were afterwards incubated until reaching an optical density (OD_600nm_) of 0.6 to 0.8 (incubation of 4–6 h at 37 °C). The expression of the poly-His tagged target protein was subsequently induced by the addition of 0.4 mM isopropyl-ß-d-thiogalactopyranoside (IPTG). After overnight incubation at 20 °C for 14 to 16 h, centrifugation at 4 °C and 5000 rpm was performed for 10 min. The resulting bacterial pellet was resuspended in lysis buffer (50 mM Tris/HCl (pH 7.6), 250 mM NaCl) with added lysozyme (1 mg/mL), phenylmethanesulfonyl fluoride (PMSF, 1 mM), and benzamidine (1 mM), and was afterwards treated with ultrasonic pulses while stored on ice. Another centrifugation was carried out for 45 min at 15.000 rpm and 4 °C. The yielded supernatant was passed through a syringe filter (pore size 0.22 µm) and submitted to a nitrilotriacetate-nickel (NTA-Ni^2+^) loaded column. After washing the column, the target protein was eluted by the application of different imidazole-containing buffers (100–500 mM). The yielded fractions were subsequently analyzed by SDS-PAGE. Fractions containing the target protein without major impurities were afterwards pooled and submitted to dialysis (50 mM Tris/HCl (pH 7.6), 100 mM NaCl, 4 °C, and constant stirring) overnight. After the addition of glycerol as a cryoprotector (final concentration of 20%), the concentration, saturating conditions of substrate and co-substrate, and the activity of *Lm*PTR1 were subsequently assessed by optical spectroscopy ([*Lm*PTR1] = 2375 µg/mL; specific activity of 0.24 U/mg *Lm*PTR1, tested in the presence of NADPH and folic acid at saturating conditions ([NADPH] = 250 µM, [folic acid] = 22.5 µM)). The yielded protein solution was stored at −80 °C in aliquots of 500 µL. The diagrams depicting the determination of the saturating conditions of folic acid and NADPH are shown in Supplementary Materials Figure S9.

### 3.9. LmPTR1-Inhibition Assay

The determination of the inhibitory activity of the in silico hits was carried out by a UV-Vis spectrophotometric assay on recombinantly produced *Lm*PTR1. In order to assess the catalytic activity of *Lm*PTR1, the oxidation of NADPH to NADP^+^ was followed with a Hitachi U-2900 UV-Vis spectrophotometer at 340 nm as a linear kinetic parameter. Reference measurements without the addition of inhibitors were used to determine the full catalytic activity (100% value), and subsequent measurements with inhibitory compounds were calculated accordingly. The total assay volume of 1 mL consisted of sodium phosphate buffer (50 mM NaH_2_PO_4_, 100 mM NaCl, pH 6), 250 µM NADPH, 35 µg *Lm*PTR1, 22.5 µM folic acid, and varying inhibitor concentrations. The tested inhibitors were dissolved in DMSO (10 mM stock solutions), and appropriate volumes were added to the assays to obtain the desired assay concentrations in such a way that a DMSO concentration between 0.1–1% resulted. No inhibition of *Lm*PTR1 could be detected by DMSO itself at the mentioned concentrations. Initial tests were performed at an inhibitor concentration of 50 µM in triplicate. In case of an inhibitory activity above 50% at this concentration, concentration-effect curves were obtained by testing four to seven different inhibitor concentrations (each of them measured in triplicate) and employing non-linear regression analysis with the software GraphPad Prism 3.0 to determine IC_50_ values (or an EC_50_ value in the case of compound **1**, due to its limited solubility in the employed assay system). The concentration-effect curves and details related to the IC_50_ determinations are presented in Supplementary Materials Figures S10–S15.

## 4. Conclusions

In the present study, we applied a two-step in silico screening (pharmacophore-based virtual screening and docking simulations) followed by in vitro enzyme inhibition tests to identify a set of new *Lm*PTR1 inhibitors out of a large collection of chemically diverse natural products. The high number of in vitro-confirmed inhibitors among the compounds identified in silico (15 active NPs out of 18 tested compounds, corresponding to a hit rate of 83%) proved the validity of the employed pharmacophore and docking-based virtual screening approach. This conclusion is additionally augmented since each of the four different pharmacophore queries employed led to the identification of NPs significantly inhibiting *Lm*PTR1. An analysis of the results of the docking simulations implied a similar pattern of interactions and binding mode of the identified inhibitors with the substrate binding site of *Lm*PTR1 comprising as an important feature π–π and H-bonding interactions with the bound co-substrate, NADPH. This hypothesis has to be evaluated by further experiments, in particular, a detailed investigation of the inhibition kinetics. Taken together, the inhibitors identified in this study may represent promising starting points for the further development of more active inhibitors of *Lm*PTR1.

## Figures and Tables

**Table 1:** Inhibitory activity of the in silico hits on *Lm*PTR1. Data represents inhibition values in % at 50 µM (n.i. = no inhibition at 50 µM), and IC_50_ values of compounds with >50% inhibition at this concentration in µM (in brackets: 95% confidence interval; *n* = 4 to 7).

Compound	% Inhibition at c = 50 µM	IC_50_ (µM)
(−)-epicatechin-3-gallate	24	
2,3-dehydrosilybin A (**1**)	66	7.3 (5.6–9.5) (EC_50_)
apigenin-7-glucoside (**2**)	60	43.5 (39.1–48.2)
eleutheroside B	12	
garcinone C (**3**)	74	26.3 (21.0–33.0)
icariside II	35	
isosakuranetin	n.i.	
isosilybin B	26	
isoxanthohumol	22	
magnolin	n.i.	
myricetin (**4**)	68	21.0 (18.3–24.2)
phillyrin	n.i.	
rosmarinic acid	20	
salvianolic acid A (**5**)	67	42.2 (38.3–46.6)
silybin A	14	
silybin B	19	
silychristin	24	
sophoraflavanone G (**6**)	90	19.2 (17.1–21.6)

## Supplementary Materials

The supplementary materials are available online.
