# Supplementary material for: In Silico Identification and In Vitro Evaluation of Natural Inhibitors of Leishmania major Pteridine Reductase I
Source: Molecules. 2017 Dec 6;22(12):2166. doi: 10.3390/molecules22122166 (PMC6149668; doi:10.3390/molecules22122166)
Supplement: Supplementary file 1 [file molecules-22-02166-s001.pdf]

molecules

Supplementary Materials

In Silico Identification and In Vitro Evaluation of Natural Inhibitors of *Leishmania major* Pteridine Reductase I

Fabian C. Herrmann [1], Nirina Sivakumar [1], Joachim Jose [2], Maria P. Costi [3], Cecilia Pozzi [4] and Thomas J. Schmidt [1,*]

[1] Institute of Pharmaceutical Biology and Phytochemistry (IPBP), University of Muenster, PharmaCampus, *Correnssstrasse* 48, D-48149 Muenster, Germany; f_herr01@uni-muenster.de (F.C.H.); nirinasiva@gmail.com (N.S.)
[2] Institute of Pharmaceutical and Medicinal Chemistry, University of Muenster, PharmaCampus, Correnstrasse 48, D-48149 Muenster, Germany; joachim.jose@uni-muenster.de
[3] Department of Life Sciences, University of Modena and Reggio Emilia, Via G. Campi 103, 41125 Modena, Italy; mariapaola.costi@unimore.it
[4] Department of Biotechnology, Chemistry and Pharmacy, University of Siena, Via A. Moro 2, 53100 Siena, Italy; pozzi4@unisi.it
* Correspondence: thomschm@uni-muenster.de; Tel.: +49-251-83-33378

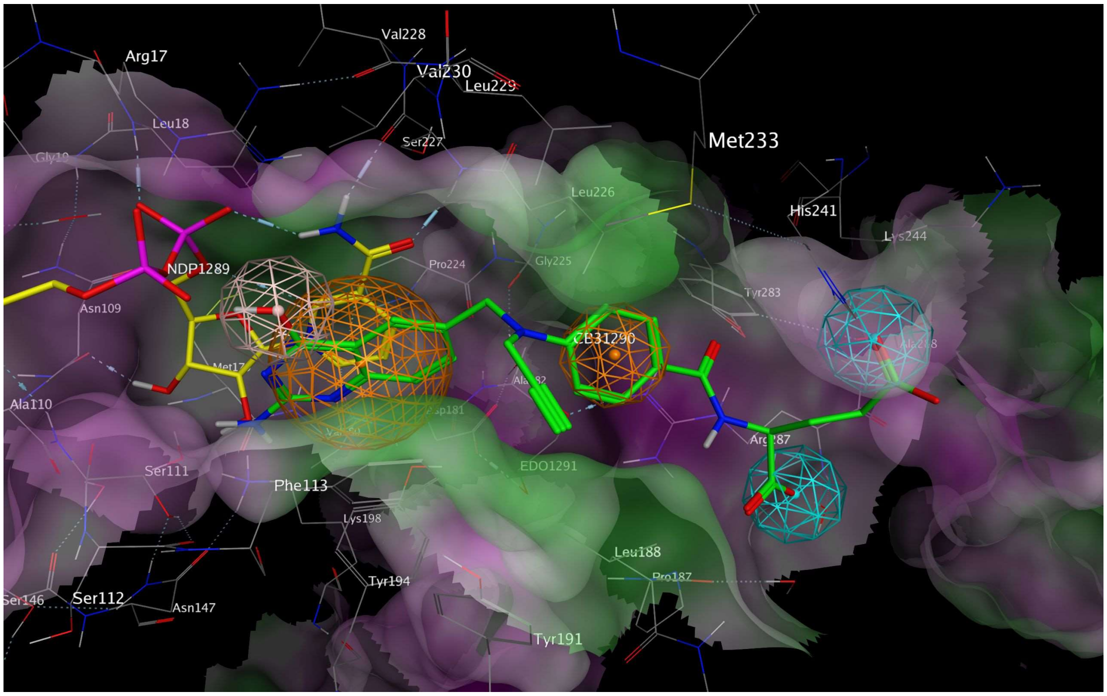

Figure S1. Pharmacophore query for *Leishmania major* pteridine reductase I (PTR1) with Protein Data Bank ID (PDB-ID) "2BFA", based on the co-crystallized inhibitor CB3. Aromatic features are displayed as orange, combined donor and acceptor features are beige-colored, H-bond acceptor features are in cyan, and exclusion spheres are not shown.

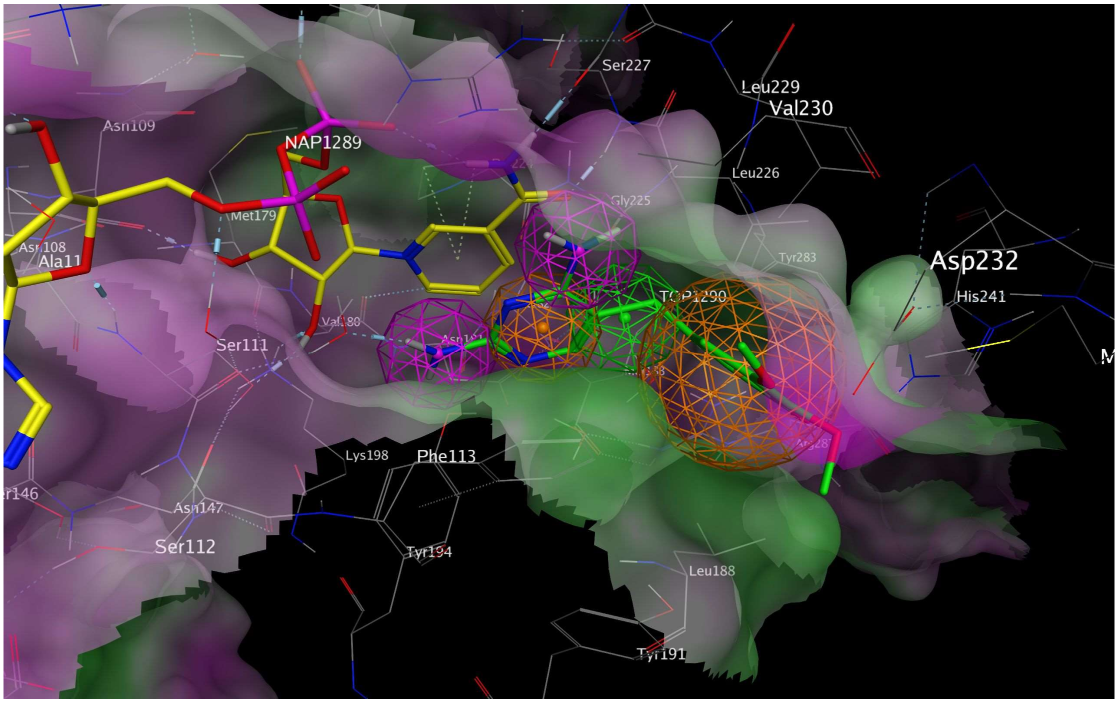

Figure S2. Pharmacophore query for *L. major* PTR1 (PDB-ID "2BFM") based on the co-crystallized inhibitor Trimethoprim. Aromatic features are displayed as orange, H-bond donor features are purple, lipophilic features are green, and the exclusion spheres are not shown.

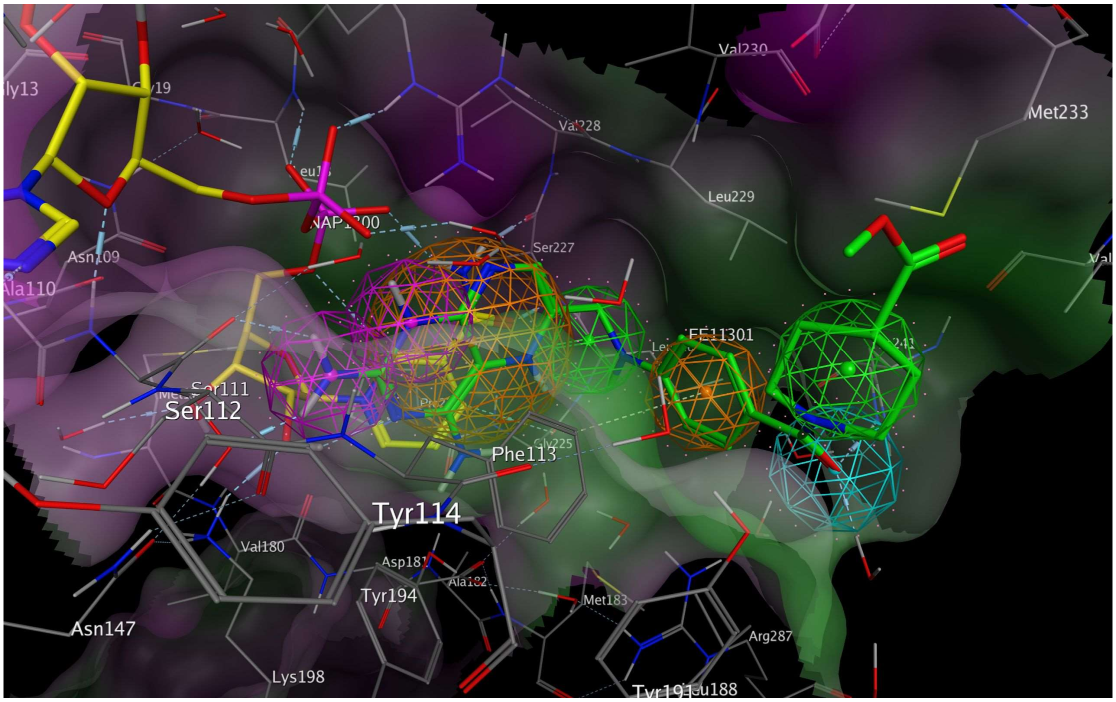

Figure S3. Pharmacophore query for *L. major* PTR1 (PDB-ID "2QHX") based on the co-crystallized inhibitor FE1. Aromatic features are displayed as orange, H-bond donor features are purple, lipophilic features are green, and the exclusion spheres are not shown.

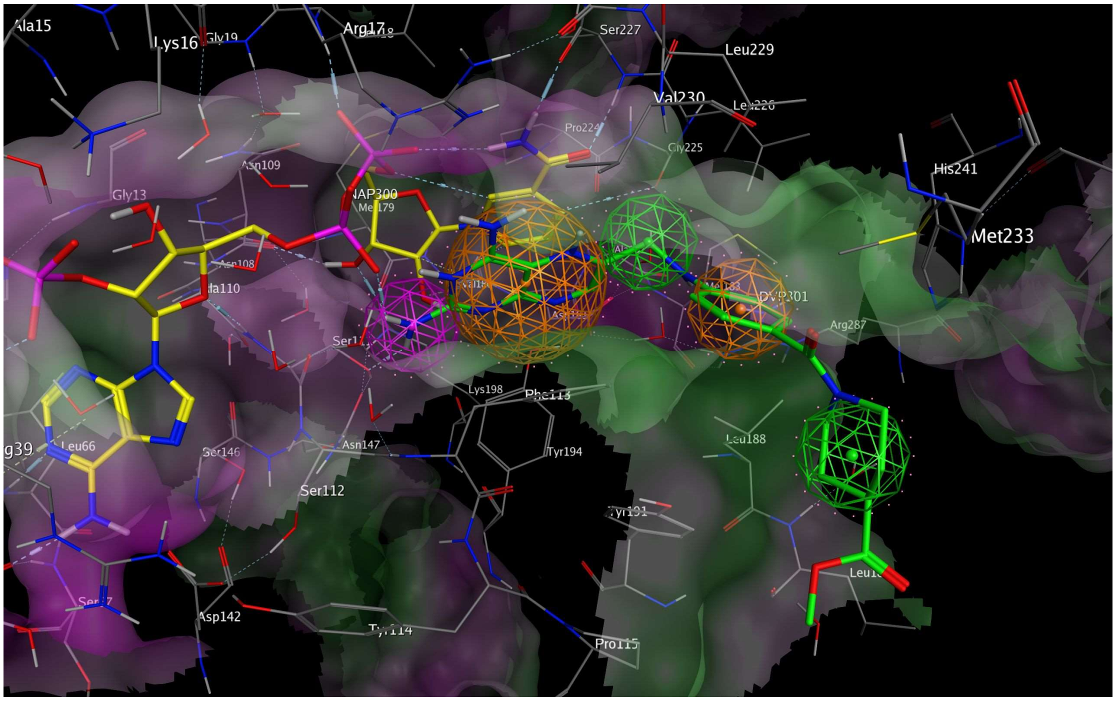

Figure S4. Pharmacophore query for *L. major* PTR1 (PDB-ID "3H4V") based on the co-crystallized inhibitor DVP; aromatic features in orange, H-bond donor features in purple, lipophilic features in green, exclusion spheres not shown.

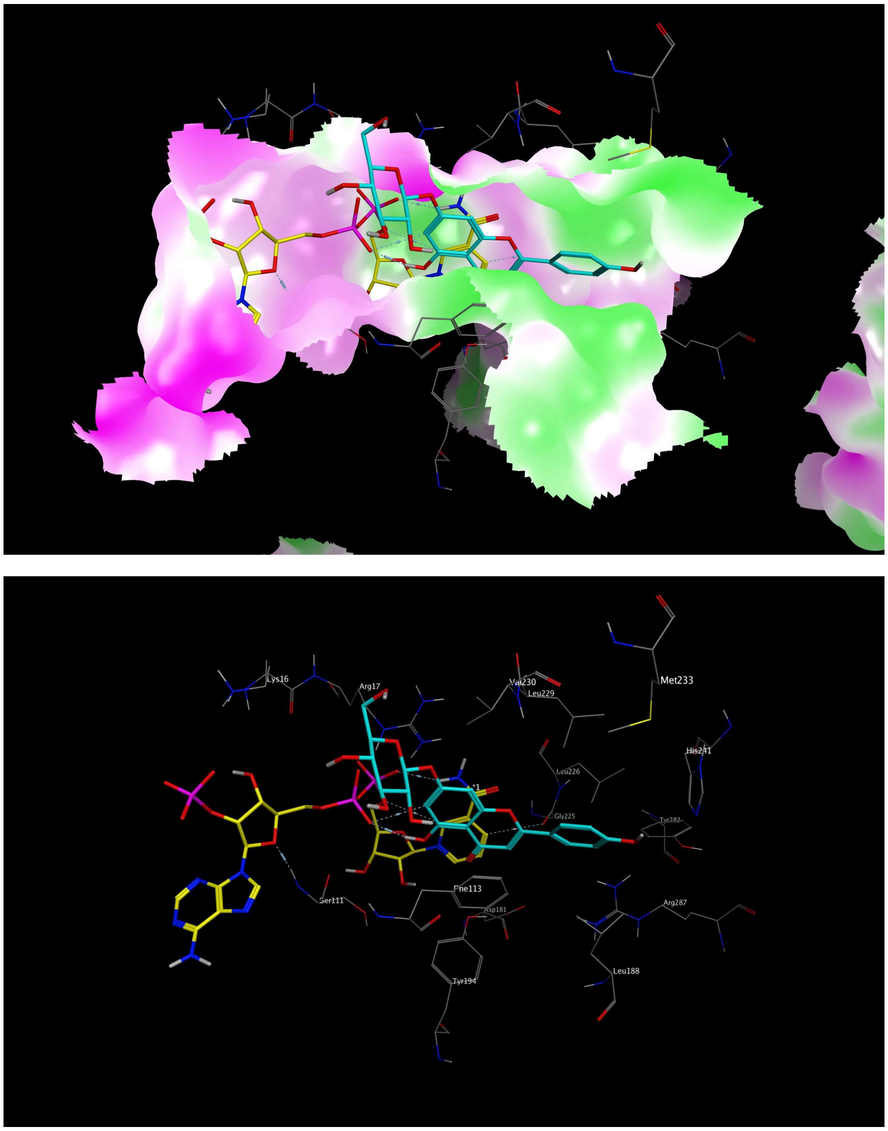

Figure S5. Best calculated docking pose of apigenin-7-glucoside (**2**) in the folic acid binding site of *Lm*PTR1 (PDB-ID "3H4V"), where co-crystallized nicotinamide adenine dinucleotide (NADP$^+$) are shown in yellow, and the best docking pose of **2** is shown in cyan. Top: The molecular surface of the binding site is colored according to lipophilicity, with green indicating high lipophilicity, and purple indicate low lipophilicity. Bottom: Surface not shown, but amino acid residues labeled.

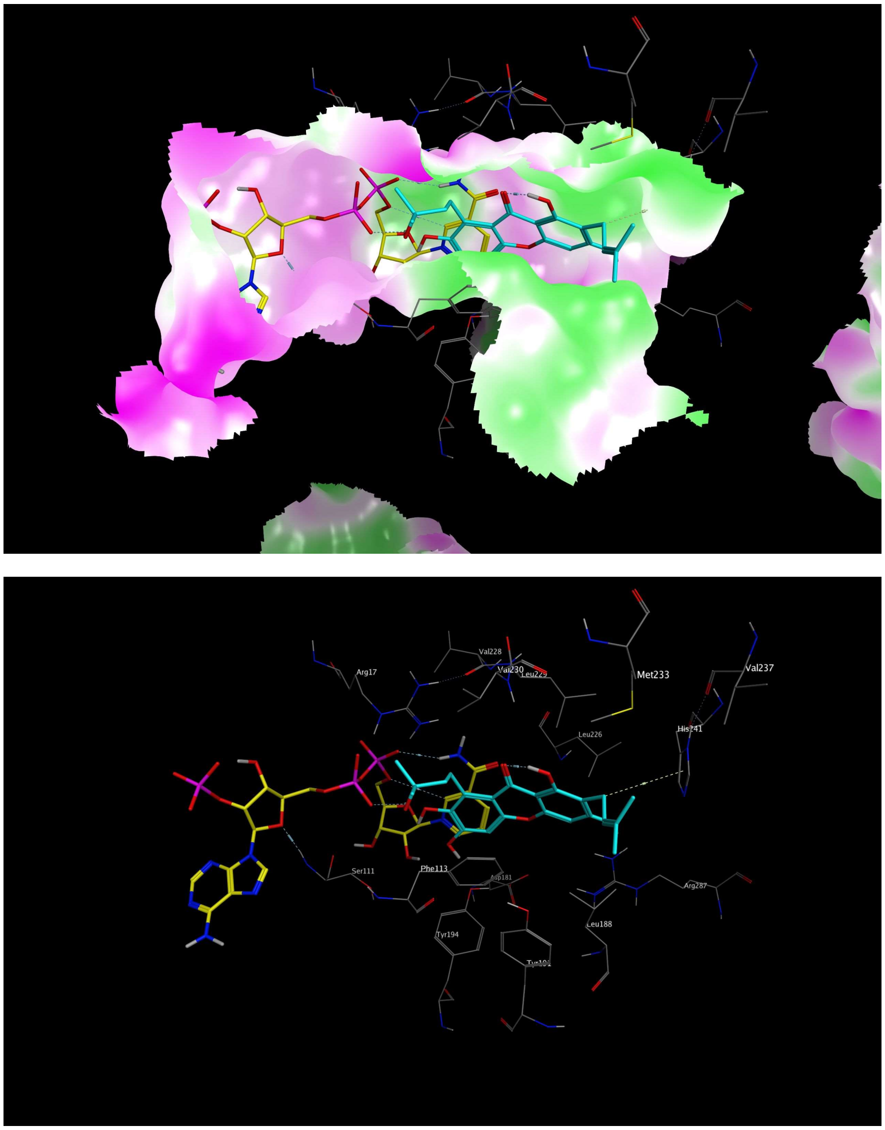

Figure S6. Best calculated docking pose of garcinone C (**3**) in the folic acid binding site of *Lm*PTR1 (PDB-ID "3H4V"), where co-crystallized $NADP^+$ are shown in yellow, and the best docking pose of **3** is shown in cyan. Top: The molecular surface of the binding site is colored according to lipophilicity, with green indicating high lipophilicity, and purple indicating low lipophilicity. Bottom: Surface not shown, but amino acid residues labeled.

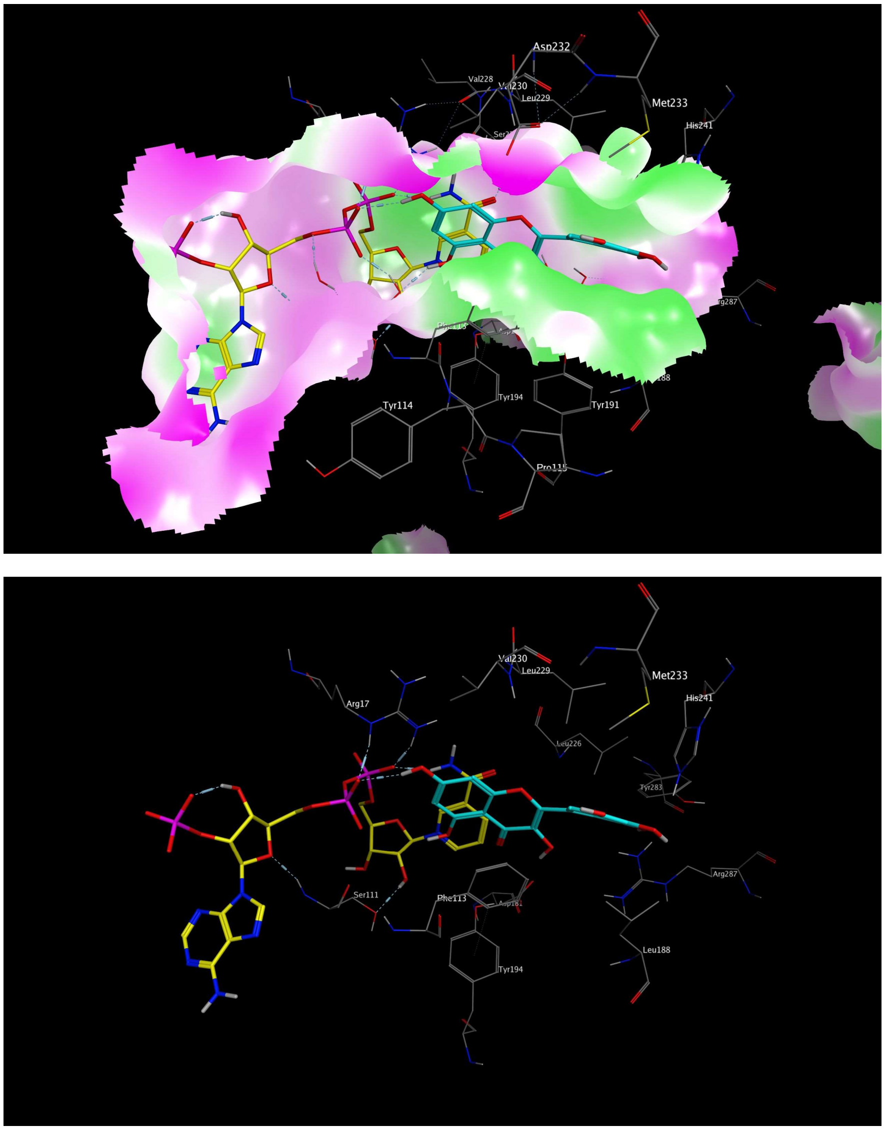

Figure S7. Best calculated docking pose of myricetin (**4**) in the folic acid binding site of *Lm*PTR1 (PDB-ID "2BFM"), with co-crystallized NADP+ shown in yellow, and the best docking pose of **4** shown in cyan. Top: The molecular surface of the binding site is colored according to lipophilicity, with green indicating high lipophilicity, and purple indicating low lipophilicity. Bottom: Surface not shown, but amino acid residues labeled.

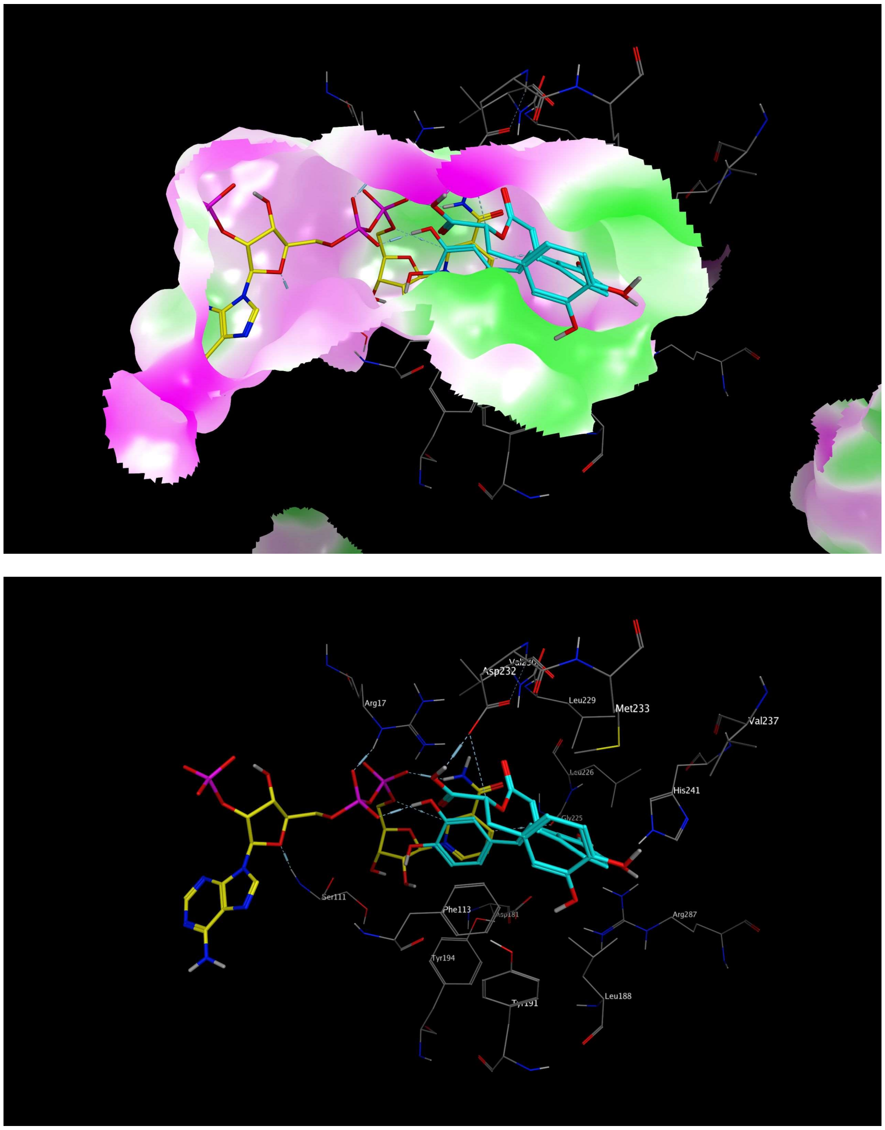

Figure S8. Best calculated docking pose of salvianolic acid A (**5**) in the folic acid binding site of *Lm*PTR1 (PDB-ID "2QHX"), with co-crystallized $NADP^+$ shown in yellow, and the best docking pose of **5** shown in cyan. Top: The molecular surface of the binding site colored according to lipophilicity, with green indicating high lipophilicity, and purple indicating low lipophilicity. Bottom: Surface not shown, but amino acid residues labeled.

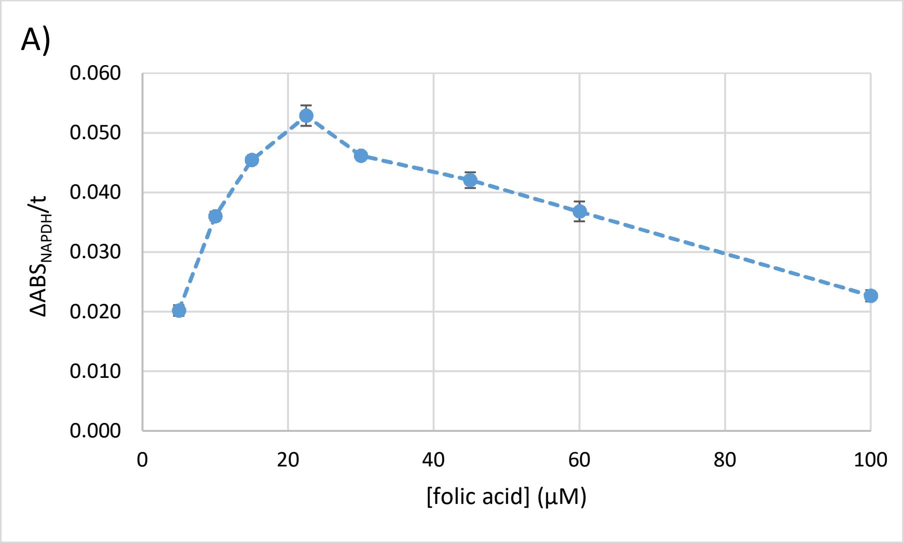

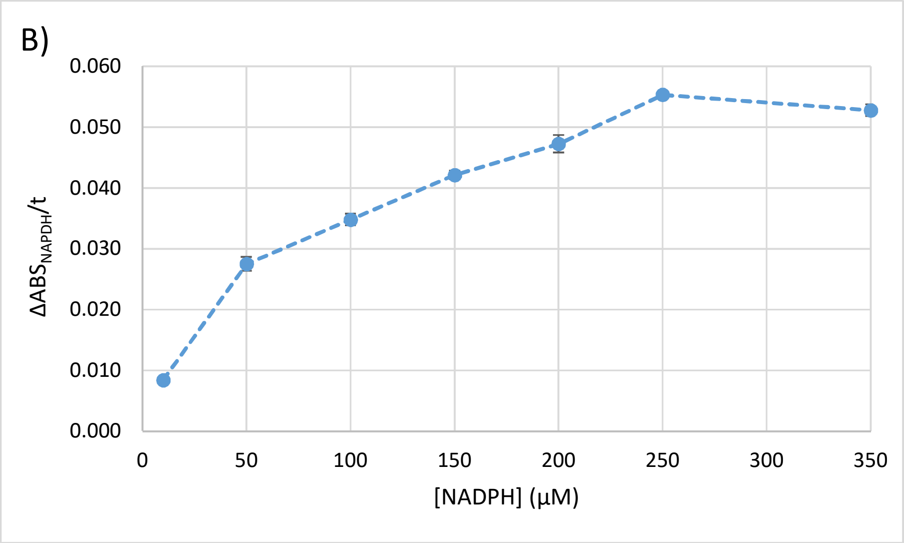

Figure S9. Experimental determination of *Lm*PTR1´s saturating conditions of folic acid and NADPH. The extent of enzymatic conversion was monitored by following the decrease of absorbance at 340 nm as a linear kinetic parameter. **(A)** Co-substrate NADPH in excess (250 μM) while varying concentrations of folic acid from 5 to 100 μM were used. The phenomenon of substrate inhibition can be clearly noticed. **(B)** Substrate folic acid at saturating conditions (22.5 μM) while varying concentrations of NADPH from 5 to 350 μM were used.

Best-fit values	
BOTTOM	19.75
TOP	56.97
LOGEC50	0.8624
HILLSLOPE	2.547
EC50	7.284
Std. Error	
BOTTOM	2.062
TOP	1.659
LOGEC50	0.05456
HILLSLOPE	0.7778
95% Confidence Intervals	
BOTTOM	15.45 to 24.05
TOP	53.51 to 60.43
LOGEC50	0.7485 to 0.9762
HILLSLOPE	0.9246 to 4.170
EC50	5.605 to 9.466
Goodness of Fit	
Degrees of Freedom	20
R²	0.9216
Absolute Sum of Squares	504.9
Sy.x	5.024

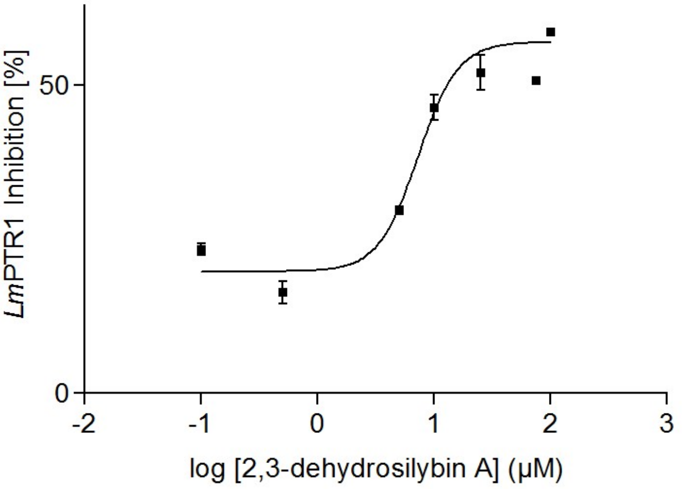

Figure S10. EC_{50} determination of 2,3-dehydrosilybin A (**1**). An IC_{50} determination could not be carried out due to the limited solubility of **1** in the employed assay system. The absolute EC_{50} value was determined by nonlinear regression analysis employing GraphPad Prism 3.00, and is given in Table 1.

Best-fit values	
BOTTOM (Constant)	0.0
TOP (Constant)	100.0
LOGEC50	1.638
HILLSLOPE	1.967
EC50	43.46
Std. Error	
LOGEC50	0.02169
HILLSLOPE	0.2154
95% Confidence Intervals	
LOGEC50	1.593 to 1.683
HILLSLOPE	1.517 to 2.418
EC50	39.14 to 48.24
Goodness of Fit	
Degrees of Freedom	19
R²	0.9669
Absolute Sum of Squares	788.9
Sy.x	6.444

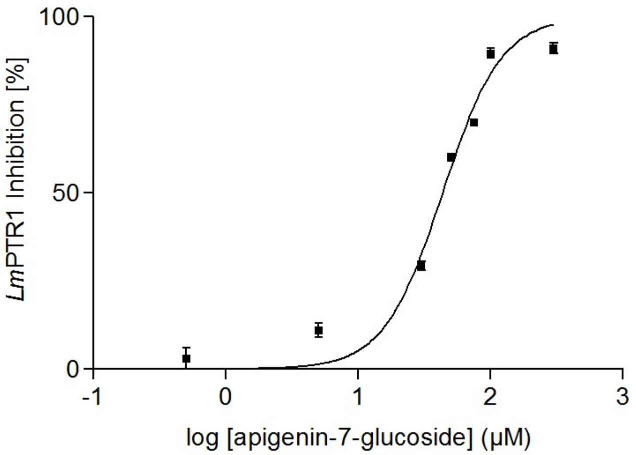

Figure S11. IC_{50} determination of apigenin-7-glucoside (**2**). The absolute IC_{50} value was determined by nonlinear regression analysis employing GraphPad Prism 3.00, and is given in Table 1.

Best-fit values	
BOTTOM (Constant)	0.0
TOP (Constant)	100.0
LOGEC50	1.420
HILLSLOPE	1.284
EC50	26.29
Std. Error	
LOGEC50	0.04645
HILLSLOPE	0.1770
95% Confidence Intervals	
LOGEC50	1.321 to 1.518
HILLSLOPE	0.9083 to 1.659
EC50	20.95 to 32.98
Goodness of Fit	
Degrees of Freedom	16
R^2	0.9174
Absolute Sum of Squares	1459
Sy.x	9.550

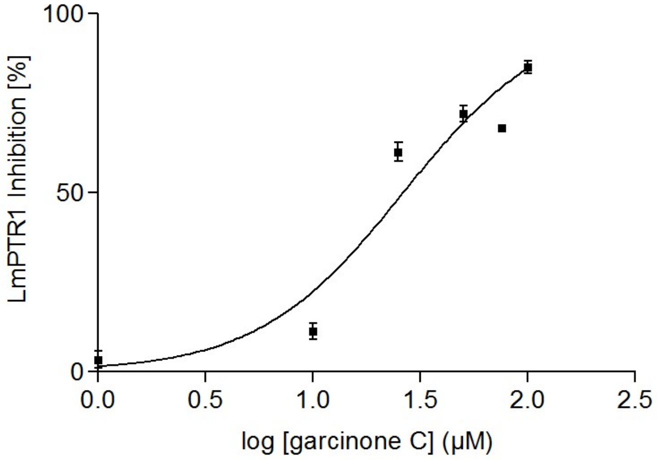

Figure S12. IC_{50} determination of garcinone C (**3**). The absolute IC_{50} value was determined by nonlinear regression analysis employing GraphPad Prism 3.00, and is given in Table 1.

Best-fit values	
BOTTOM (Constant)	0.0
TOP (Constant)	100.0
LOGEC50	1.322
HILLSLOPE	1.312
EC50	21.01
Std. Error	
LOGEC50	0.02807
HILLSLOPE	0.1163
95% Confidence Intervals	
LOGEC50	1.262 to 1.383
HILLSLOPE	1.061 to 1.563
EC50	18.27 to 24.15
Goodness of Fit	
Degrees of Freedom	13
R^2	0.9716
Absolute Sum of Squares	382.3
Sy.x	5.423

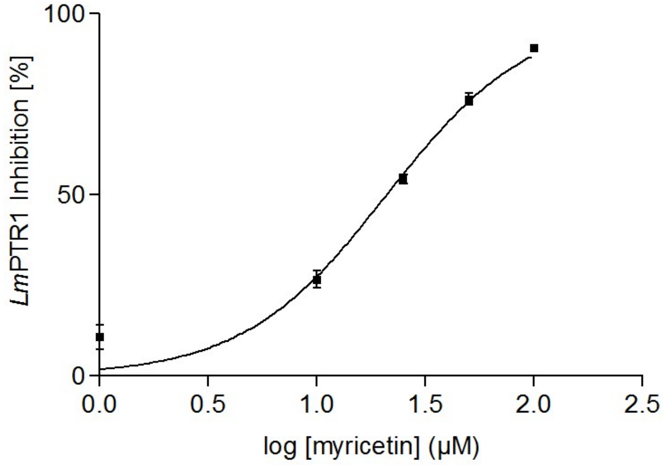

Figure S13. IC_{50} determination of myricetin (**4**). The absolute IC_{50} value was determined by nonlinear regression analysis employing GraphPad Prism 3.00, and is given in Table 1.

Best-fit values	
BOTTOM (Constant)	0.0
TOP (Constant)	100.0
LOGEC50	1.626
HILLSLOPE	1.832
EC50	42.23
Std. Error	
LOGEC50	0.02046
HILLSLOPE	0.1519
95% Confidence Intervals	
LOGEC50	1.583 to 1.668
HILLSLOPE	1.514 to 2.150
EC50	38.26 to 46.60
Goodness of Fit	
Degrees of Freedom	19
R²	0.9736
Absolute Sum of Squares	644.4
Sy.x	5.824

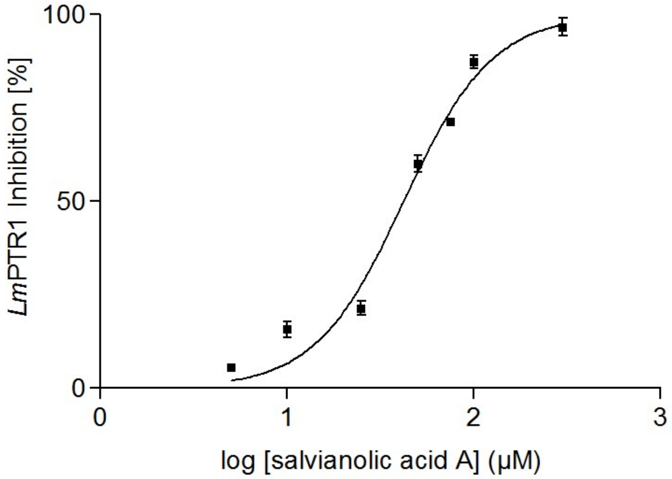

Figure S14. IC_{50} determination of salvianolic acid A (**5**). The absolute IC_{50} value was determined by nonlinear regression analysis employing GraphPad Prism 3.00, and is given in Table 1.

Best-fit values	
BOTTOM (Constant)	0.0
TOP (Constant)	100.0
LOGEC50	1.284
HILLSLOPE	2.201
EC50	19.24
Std. Error	
LOGEC50	0.02377
HILLSLOPE	0.1983
95% Confidence Intervals	
LOGEC50	1.234 to 1.335
HILLSLOPE	1.781 to 2.621
EC50	17.14 to 21.61
Goodness of Fit	
Degrees of Freedom	16
R²	0.9675
Absolute Sum of Squares	688.7
Sy.x	6.561

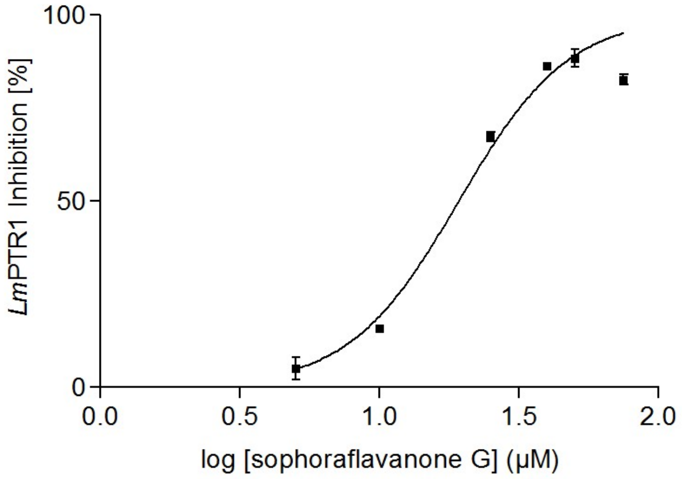

Figure S15. IC_{50} determination of sophoraflavanone G (**5**). The absolute IC_{50} value was determined by nonlinear regression analysis employing GraphPad Prism 3.00, and is given in Table 1.
